# Thermal Study of Carbon-Fiber-Reinforced Polymer Composites Using Multiscale Modeling

**DOI:** 10.3390/ma16227233

**Published:** 2023-11-19

**Authors:** Wiem Nasri, Zied Driss, Ridha Djebali, Kyu-Yeon Lee, Hyung-Ho Park, Abderazak Bezazi, Paulo N. B. Reis

**Affiliations:** 1 Laboratory of Electro-Mechanic Systems (LASEM), National School of Engineers of Sfax (ENIS), University of Sfax (US), B.P. 1173, Road Soukra km 3.5, Sfax 3038, Tunisia; zied.driss@enis.tn; 2 UR22ES12: Modeling, Optimization and Augmented Engineering, ISLAI Béja, University of Jendouba, Béja 9000, Tunisia; ridha.djebali@islaib.u-jendouba.tn; 3 Department of Materials Science and Engineering, Yonsei University, Seoul 03722, Republic of Korea; dwrky@yonsei.ac.kr (K.-Y.L.); hhpark@yonsei.ac.kr (H.-H.P.); 4 Laboratory of Applied Mechanics of New Materials (LMANM), Université 8 Mai 1945—Guelma, B.P. 401, Guelma 24000, Algeria; ar_bezazi@yahoo.com; 5 University of Coimbra, CEMMPRE, ARISE, Department of Mechanical Engineering, 3030-788 Coimbra, Portugal

**Keywords:** carbon-fiber-reinforced polymer, multi-scale modeling, micro-scale, woven fabric, thermal conductivity

## Abstract

The layered fibers of carbon-fiber-reinforced polymer (CFRP) composites exhibit low thermal conductivity (TC) throughout their thickness due to the poor TC of the polymeric resin. Improved heat transmission inside the hydrogen storage tank during the filling process can reduce further compression work, and improved heat insulation can minimize energy loss. Therefore, it is crucial to understand the thermal properties of composites. This paper reports the thermal behavior of plain-woven CFRP composite using simulation at the micro-, meso-, and macro-scales. The TC was predicted numerically and compared to experimental findings and analytical models. Good results were found. Using the approach of multi-scale modeling, a parametric study was carried out to analyze in depth the influence of certain variables on thermal properties. The study revealed that both fiber volume fraction and temperature significantly influenced the TC of the composite, with the interphase fiber/matrix thickness following closely in terms of impact. The matrix porosity was found to have a relatively slighter impact, particularly within the porosity range of 5 to 15%.

## 1. Introduction

Composite materials reinforced with carbon fiber are relatively widespread, and their application is increasing. These high-performance composites are lightweight, robust, and resistant to corrosion. Beyond these inherent qualities, carbon-fiber-reinforced polymer (CFRP) distinguishes itself through exceptional fatigue and creep resistances, particularly when subjected to harsh environmental conditions and varying loads [1,2,3,4,5,6]. These unique attributes position CFRP as a superior choice among fiber-reinforced polymers (FRPs). In the realm of applications, CFRP is at the forefront of diverse industries, playing a pivotal role in aerospace, automotive, construction, and sports equipment. Its prevalence in these sectors underscores its versatility and underscores the material’s significant impact on advancing technology and engineering solutions. Most composites developed in previous years were designed and manufactured to enhance mechanical features such as toughness, stiffness, and strength. However, high thermal capacity, especially the ability to endure high temperatures and remove heat quickly from thermal sensorial equipment or heat exchangers, is becoming extremely relevant. In the literature, there is a wealth of studies on the mechanical characteristics of composites. Thermal assets, on the other hand, are addressed in fewer accounts. Because the heat-conduction qualities of carbon may be modified to fit the demands of a specific engineering application, it is among the most adaptable materials for thermal control. Thermal conductivity (TC) is increasingly being given more thought in order to broaden the applications of composites. When reinforced polymer composites are employed, they face several challenges. In fact, they are frequently subjected to a quickly changing temperature setting. As a result, thermal stress is created, which has considerable influence on composite stiffness and strength [7]. Considering that polymer composites are frequently thin, the simplest way to eliminate heat from a composite structure is through its thickness direction. Nonetheless, Shim et al. [8] reported that the ratio of transverse to in-plane thermal conductivities may be adjusted from 30 to 130, restricting heat transport in the thickness direction. The thermal stress generated by temperature changes has been shown to have a significant influence on material failure [9,10,11,12]. As a result, it is critical and necessary to explore the heat conductivity of composites.

Many researchers have considered various techniques for predicting the heat transfer and thermal properties of such composites. Most anisotropic composite material research has concentrated on mechanical properties, with just a few models developed to estimate heat conductivity characteristics in several directions. Mechanical parameters that have been experimentally established can be validated using recently reported analytical models. However, there are just a few models for TC that are available for casual verification. Most studies predict effective thermal conductivity (ETC) across a material’s thickness. Using the law of mixtures, however, heat conductivity along a composite’s fiber orientation may be easily anticipated. Models that predict transverse conductivity are mostly dependent on the geometrical configuration of the reinforcement in the investigation. The unidirectional reinforcement models of Charles and Wilson [13] and Maxwell [14] were employed. A crimp angle was included with these models to account for the undulation phenomena of the analyzed woven fabric. Ning and Chou’s model [15] was employed to estimate the transversal TC of braided composites. However, these models are valid if the effective TC is independent of temperature. This is not the case with this study. TC is believed to be constant in ordinary problems. Nonetheless, when the temperature difference is significant and coupled with a change in characteristics, the influence of temperature on TC must be considered while developing a practical problem. Currently, the mathematical formulation of heat conduction related to temperature-dependent TC is described as nonlinear. The strong superposition concepts of linear theory cannot be employed to develop analytical solutions. Some researchers were able to provide analytical solutions for the non-linearity of heat conduction problems, but they had to assume material homogeneity. Chang and Payne [16], for instance, provided an analytical expression for heat conduction in a two-layered block with linear temperature-dependent TC using the Kirchhoff transformation. Many additional studies concentrated on constructing predictive models and performing tests to assess the heat conductivity of unidirectional and particle composites.

So far, all analytical solutions have either concentrated on geometrical variables while ignoring the role of temperature on thermal properties, or they assume material homogeneity and ignore the influence of geometrical factors. With the advancement of computer computational capabilities, finite element analyses (FEA) have become frequently employed in the numerical computation of heat conduction. Li et al. [17] determined both the in- and out-of-plane thermal conductivities of woven composites using the representative volume element (RVE) technique with two-unit cells created at varied scales and periodic boundary conditions. Ran et al. [18] created a numerical model to assess combined heat and mass transmission in woven fibrous composites while taking geometrical properties into account. Matusiak [19] established a model of woven fabric thermal resistance based on a square cross-section of yarn. Rafiee and Salehi [20,21] and Yazdanparast and Rafiee [22] presented two-scale and multiscale studies using “bottom-up” and “top-down” approaches to predict the burst pressure of a wound composite vessel while taking into consideration the influence of numerous factors on composite failure. Also, different types of RVE shapes were discussed. It was discovered that fiber packing has little impact on the mechanical characteristics of micro-scale RVE and that fiber contiguity and spacing have a significant impact. Even though these approaches can accurately compute the effective properties of woven composites, there is insufficient FEA research dealing with the influence of micro voids, cracks, and internal defects on the thermal properties of CFRP at the microscale level. During the early preparation of composites, flawed interfaces are frequently produced because of chemical reactions, heat treatment, and interfacial debonding, among other factors. This has a significant effect on the thermomechanical properties of the composite. It has been shown that the tensile stresses and potential cracks in woven composite engineering structures typically start in the microstructure when subjected to mechanical loads, cyclic internal pressure, or harsh environments. Progressive failure begins with the introduction of voids in CFRP. Neglecting these facts would be harmful to the regular review of industrial applications, especially when using CFRP for storing hydrogen in gaseous high-pressure vessels or applications where the composite is facing cyclic load change.

While there have been numerous studies on the mechanical behavior of CFRPs, there has been relatively less research on their thermal behavior. Most existing thermal studies are empirical in nature and the numerical studies that do exist often overlook important variables that affect thermal behavior. One key novelty of the present work is the incorporation of temperature effects, which is essential due to the nonlinear nature of CFRP composites under varying thermal conditions. By considering temperature-dependent behavior, it provides a more comprehensive understanding of how thermal factors influence the composite’s overall performance. Furthermore, various factors that significantly impact its thermal behavior were incorporated. These factors include temperature, fiber volume fraction, matrix porosity (voids), and interphase thickness. Moreover, two different coating materials were used for the interphase layer, acknowledging the influence of material choice on the thermal properties. Overall, our proposed extended finite element analysis not only advances the understanding of CFRP’s thermal behavior but also has the potential to enhance design considerations and practical applications in real-world industrial scenarios. By incorporating multiple influential factors and overcoming the limitations of previous studies, it contributes to a more comprehensive understanding of CFRP composites and their thermal behavior.

Regarding this issue, this paper proposes to conduct an extended finite element analysis of heat transfer of CFRP woven fabric composites to determine the effective thermal conductivities of composites using both unit cells with various length scales. The content of this paper can be outlined as follows. First, unit cells are built in two sizes and periodic boundary conditions are applied to estimate the thermal conductivities in the in-plane and through-thickness directions of the unit cells. The model is then validated. Those results could also be applied to unidirectional (UD) composites with fiber reinforcement. The calculated output material characteristics of the unit cell design are then employed as input for the macro-scale model. Consequently, the thermal properties of the whole composite can be computed. Following the establishment of the fundamental framework of the multi-scale model for CFRP, a parametric study is performed to evaluate the influence of various factors such as temperature, fiber volume fraction, matrix porosity, and interphase fiber/matrix thickness on the microscale level. The final part presents and summarizes the study’s findings.

## 2. Methodology

**Figure 1:**
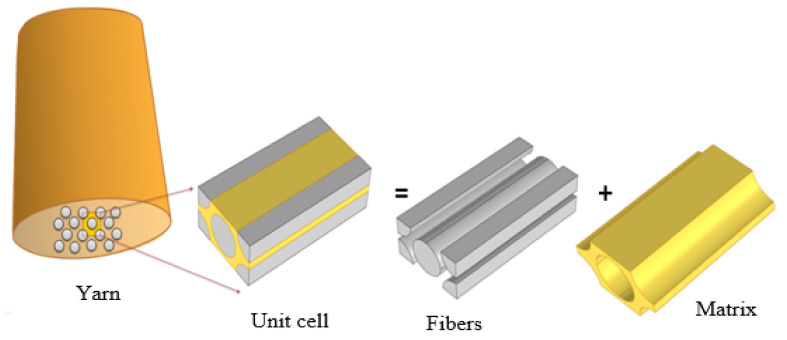
Micro-scale hexagonal unit cell model: unit cell 1.

It is necessary to measure the material characteristics of each individual lamina in order to assess components constructed from laminated composites. Micromodeling analysis involves employing unit cells to compute the homogenized material properties of a lamina. The homogenized material properties can be calculated if the material properties and distribution of the fibers and matrix are known. Composite materials are commonly periodic micro-structured, such as carbon fibers. In order to reduce computational costs, simulations of a composite material with a homogeneous domain with equivalent properties are used. The UD laminas may be employed to forecast the thermal properties of complicated fabric structural composite materials using the reduced unit-cell model. The smallest volume element of a lamina, known as RVE, is the tiniest volume element that may be repeated to define the whole lamina. A lamina is made up of fibers and matrix in general. The matrix material is isotropic, while the fibers are frequently orthotropic. In this work, the following material properties are computed from a selected unit cell shape. The material properties at room temperature are mentioned in Table 1 [23]. The meso-microscopic scale is represented by unit cell 1. The latter was built within COMSOL Multiphysics (version 5.2a) and includes one fiber and four 1/4 fibers. The dimensions of the unit cell are 0.38 × 0.22 × 0.8 mm as sketched in Figure 1.

**Figure 2:**
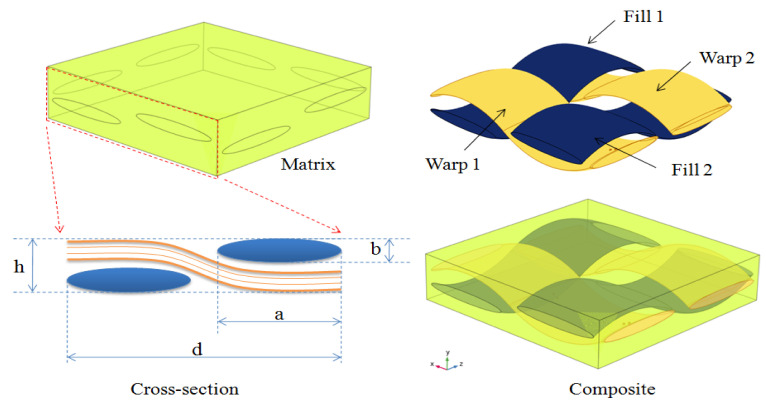
The mesoscale unit cell of plain-woven composite: unit cell 2.

**Figure 3:**
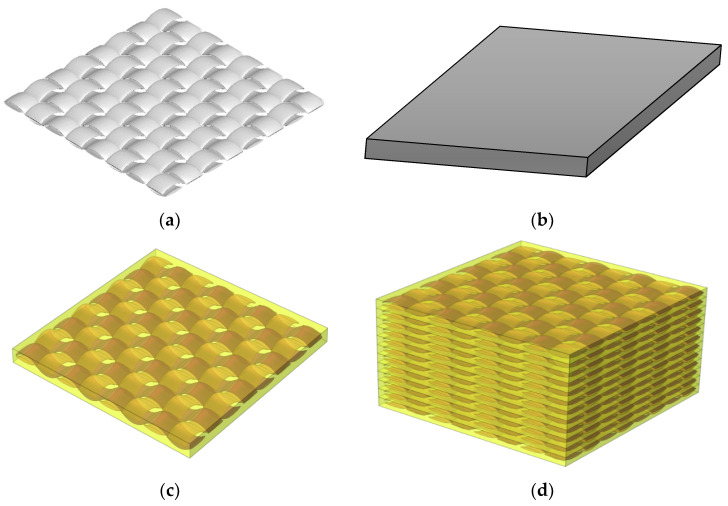
Macro-scale composite model: (**a**) woven fabric; (**b**) matrix; (**c**) 1-layered composite; (**d**) 12-layered composite.

The fiber size in the tow is determined so that the volume fraction of the fiber and the external diameter of the tow are proportional to those of a real tow. According to the literature, a real genuine carbon fiber reinforced composite is transversely isotropic which is maintained by hexagonal packing because of the random distribution of fibers along the cross-section of the yarn [17]. Therefore, the inculcated fibers inside the yarns are supposed to be distributed in hexagonal arrays. There are hundreds of fibers in a single yarn with a random distribution. The shape of the fibers is circular with a reasonably consistent diameter when viewed in cross-section. It is reasonable to imagine the yarns in a woven composite as a UD fiber-reinforced composite, since the scale of yarn undulations is often considerably larger than that of carbon fiber diameter. For the macro–mesoscopic scale, unit cell 2 is shown in Figure 2 and the impact of waviness is considered. Fabric-reinforced composites can be made by weaving together fluctuated warp and weft threads and afterward impregnating them with epoxy resin. To ensure that the fiber volume portion remained relatively stable, and the characteristics were unaffected by position, the cross-section form of yarns is intended to be elliptical and fixed. Figure 3 presents the macro-scale model of the laminated composite which was created using SolidWorks software (version 16.0). Both unit cells depend on translational symmetry. All plies can be considered similar and can be replicated by another with a simple translation. To better explain the design of the composites, the unit cell approach was used. For the 1-layered composite, the dimensions were obtained by replicating the unit cell in the x and y directions, four times. This approach ensured that the composite had consistent dimensions and allowed for a representative analysis of the composite structure. Similarly, the 12-layered composite replicated the 1-layered composite 12 times in the z direction or through the thickness direction. This stacking of multiple identical layers allowed us to create a composite with enhanced properties while maintaining a consistent design. Hence, the design of the composites involved replicating unit cells to establish the dimensions and stacking multiple layers to create the desired composite architecture. The assumption of similar and replicable layers allowed for a focused analysis on a single layer, while accurately representing the overall behavior of the composites.

The fiber volume fraction V_y_ in unit cell 1 is calculated as:

(1) $$V_{y}=\frac{4\;\pi \;a\;b\;l}{h\;d^{2}}$$

where a and b are the elliptical cross section’s long and short diameters, respectively, and l denotes the yarn’s length. The mesoscale model’s side length and height are d and h. Table 2 summarizes the fundamental parameters. The fiber packing ratio in the yarn V_fy_ represents the dispersed composition of fibers in an individual yarn. It is a crucial determinant when calculating the dimensions of the micro-scale model. The volume fraction of the whole composite V_f_ is expressed as follows:

(2) $$V_{f}=V_{fy}\;V_{y}$$

To consider the correlation between micro-scale, meso-scale, and macro-scale models in simulations, a multiscale approach is employed. This approach aims to capture the behavior of composite materials at different length scales and integrate them into a cohesive simulation framework. At the micro-scale, the behavior of individual constituents, such as fibers, matrix, and interphase, is analyzed. This can be done through detailed finite element analysis (FEA). By examining the micro-scale behavior, important information regarding thermal conductivity, thermal diffusivity, and other properties can be obtained. Moving to the meso-scale, the focus shifts to the organization and arrangement of the constituents at a larger length scale, such as the woven fabric structure or laminate configuration. The effective properties derived from the micro-scale analysis are incorporated into an RVE or unit cell. This meso-scale model plays a crucial role in capturing the macroscopic behavior of the composite, including thermal transport and mechanical response. Finally, at the macro-scale, the behavior of the composite material at the system or component level is considered. This involves taking into account the overall geometry, loading conditions, and boundary conditions. By integrating the information from the meso-scale model, the macro-scale model provides predictions of the composite’s global behavior, such as temperature distribution, thermal conductivity, and other macroscopic properties. To establish the correlation between these models, information flows from the micro-scale to the meso-scale and then to the macro-scale. The effective properties obtained from the micro-scale analysis, such as thermal conductivity and diffusivity, serve as inputs for the meso-scale model. The meso-scale model, in turn, provides the effective properties of the composite, which are then used as inputs for the macro-scale model. Maintaining consistency and accuracy in transferring information between the scales is crucial. Proper homogenization techniques can be employed to determine effective properties at each scale. Additionally, the validation and calibration of the models against experimental data at different scales are essential for establishing correlation and ensuring the predictive capability of the simulation framework. In summary, the correlation between micro-scale, meso-scale, and macro-scale models in simulations is achieved by integrating information and properties obtained at each scale. This multiscale approach allows for a comprehensive understanding of the material behavior and enables accurate predictions of the composite’s performance. Before carrying out heat transfer analysis, the following hypotheses are presumed. First, the fibers inside the yarn are distributed in a regular pattern with no overlap. The yarn is made of untwisted UD fibers joined together by epoxy resin. Second, the yarns in the woven fabric are arranged in a periodic pattern. The interwoven yarns are assembled and bonded with resin to constitute a layer. Finally, in order to validate our models primordially, we assumed that the interface fiber/matrix is properly bonded with the strength of the epoxy resin and that there are no micro voids in the matrix. In other words, the simulation assumed an ideal scenario where the fiber/resin interface was perfectly bonded, implying an interphase thickness of 0. This assumption allowed to establish a baseline for the interfacial bonding performance in order to validate the model with the work of Dong et al. [23]. Following this, variations were systematically introduced by incrementally increasing the interphase thickness. This step was taken to simulate the presence of an additional layer between the matrix and the fiber. The purpose of this increment was to explore how changes in the interphase thickness influence the overall interfacial bonding characteristics. By introducing this layered approach, we aimed to capture the nuanced effects of interfacial interactions. As the interphase thickness increased, it represented a more realistic depiction of the actual physical conditions within fiber-reinforced composites. This iterative process allowed us to observe and analyze the evolving behavior of the interface under different interphase thicknesses. The impact of matrix porosity and interphase thickness at the microscopic level, with two carbon fiber coating materials, will be developed in Section 5 along with the effect of temperature and fiber volume fraction.

## 3. Numerical Models

### 3.1. Governing Equations

Heat conduction FEA were performed using the commercially available finite element software COMSOL Multiphysics (version 5.2a). The finite element computations of micro-scale and meso-scale models were carried out in a steady state. The governing equation is described as:

(3) $$\lambda_{xx}\;\frac{\partial^{2}\;T}{\partial \;x^{2}}+\lambda_{yy}\;\frac{\partial^{2}\;T}{\partial \;y^{2}}+\lambda_{zz}\;\frac{\partial^{2}\;T}{\partial \;z^{2}}+(\lambda_{xy}+\lambda_{yx})\;\frac{\partial^{2}\;T}{\partial \;x\;\partial \;y}+(\lambda_{xz}+\lambda_{zx})\;\frac{\partial^{2}\;T}{\partial \;x\;\partial \;z}+(\lambda_{yz}+\lambda_{zy})\;\frac{\partial^{2}\;T}{\partial \;y\;\partial \;z}=0$$

while the FEA of the full-scale model was run for transient thermal conditions as described in the following equation:

(4) $$\lambda_{xx}\;\frac{\partial^{2}\;T}{\partial \;x^{2}}+\lambda_{yy}\;\frac{\partial^{2}\;T}{\partial \;y^{2}}+\lambda_{zz}\;\frac{\partial^{2}\;T}{\partial \;z^{2}}+(\lambda_{xy}+\lambda_{yx})\;\frac{\partial^{2}\;T}{\partial \;x\;\partial \;y}+(\lambda_{xz}+\lambda_{zx})\;\frac{\partial^{2}\;T}{\partial \;x\;\partial \;z}+(\lambda_{yz}+\lambda_{zy})\;\frac{\partial^{2}\;T}{\partial \;y\;\partial \;z}=C_{p}\;\rho \;\frac{\partial \;T}{\partial \;t}$$

where T is the temperature, λ_xi_, λ_yi,_ and λ_zi_ are the three lines of the TC tensor, and i is a direction among x, y, and z. The carbon fiber is considered to be transversely isotropic, and the resin matrix is isotropic. The specific heat and density, correspondingly, are C_p_ and q. The constitutive relations used in FEA are identical to those found in Fourier’s law of heat conduction and are listed below:

(5) q=−λ ∇T

### 3.2. Boundary Conditions

**Figure 4:**
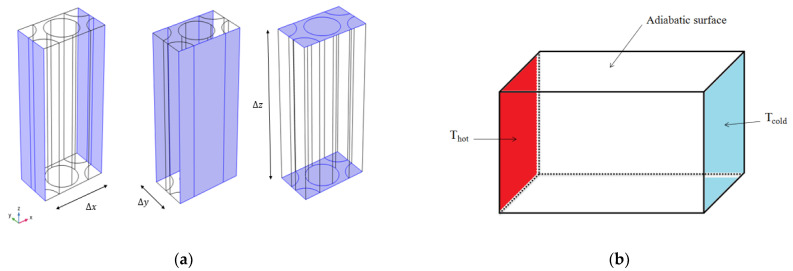
Boundary conditions: (**a**) Periodic boundary conditions; (**b**) macro boundary conditions.

To preserve the consistency of the temperatures of the relevant nodes on the parallel sides, periodic temperature boundaries were employed. At the opposing boundaries of the three directions x, y, and z, a temperature offset is applied. The temperature profiles at these opposing borders are not necessarily homogeneous, but they are pointwise equal up to a constant offset of 1 K. The fibers and nearby unit cells should not be separated or overlapped. Periodic boundary conditions (PBCs) were designed by Xia et al. [24] and improved by Li et al. [25]. Figure 4a shows the PBCs applied for the micro-scale unit cell model. The ETC for the macro-scale model is estimated by implementing a thermal gradient along the thickness direction and supposing the remaining surfaces are adiabatic (Figure 4b).

### 3.3. Meshing

**Figure 5:**
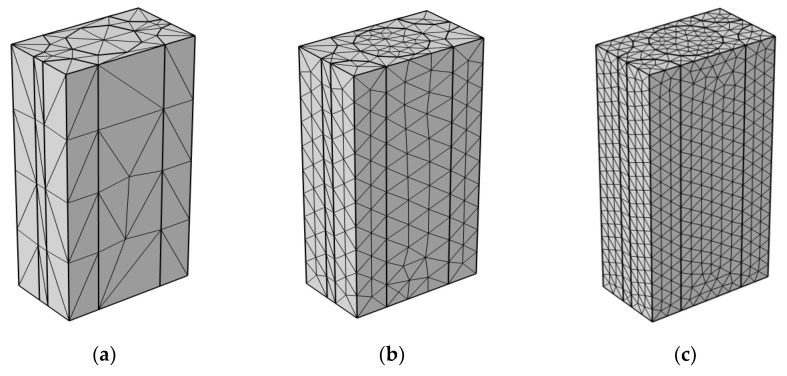

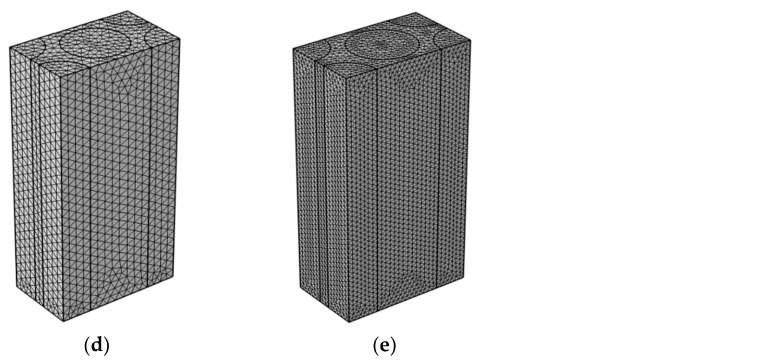
Meshing for micro-scale unit cell model: (**a**) extra coarse; (**b**) normal; (**c**) finer; (**d**) extra fine; (**e**) extremely fine.

COMSOL Multiphysics was used to generate a triangular mesh. Following pre-testing with various mesh sizes, the most appropriate mesh is chosen as “Finer”. For the micro-scale unit cell model, after confirming that additional refining would not change the solution, an optimal mesh density of 15,492 elements is obtained as shown in Table 3. As for meso- and macro-scale models, the optimal number of elements is 475,222 and 1,590,741, respectively. The mesh distribution for the micro-scale unit cell model is shown in Figure 5.

## 4. Models Validation

### 4.1. Micro-Scale Model Validation

To confirm the accuracy of the micro-scale TC computation, mathematical models tailored to UD fiber-reinforced composites were used with the basic thermal characteristics of the fiber and matrix employed, i.e., carbon fiber and epoxy resin. The thermal conductivities of the yarns at an ambient temperature equal to T = 25 °C are then calculated. The expression of the yarn’s axial TC λ_ya_ for the parallel model [23] is written as:

(6) $$\lambda_{ya}=\lambda_{f\;∥}\;V_{fy}+\lambda_{m}\;(1-V_{fy})$$

The yarn’s transversal TC λ_yt_ for the Pilling model [26] is expressed as follows:

(7) $$\lambda_{yt}=\lambda_{m}\;{\left[\frac{\sqrt{{\left(1-V_{fy}\right)}^{2}\;{\left(\frac{\lambda_{f⊥}}{\lambda_{m}}-1\right)}^{2}+4\;\frac{\lambda_{f⊥}}{\lambda_{m}}}-(1-V_{fy})\;\left(\frac{\lambda_{f⊥}}{\lambda_{m}}-1\right)}{2}\right]}^{2}$$

**Figure 6:**
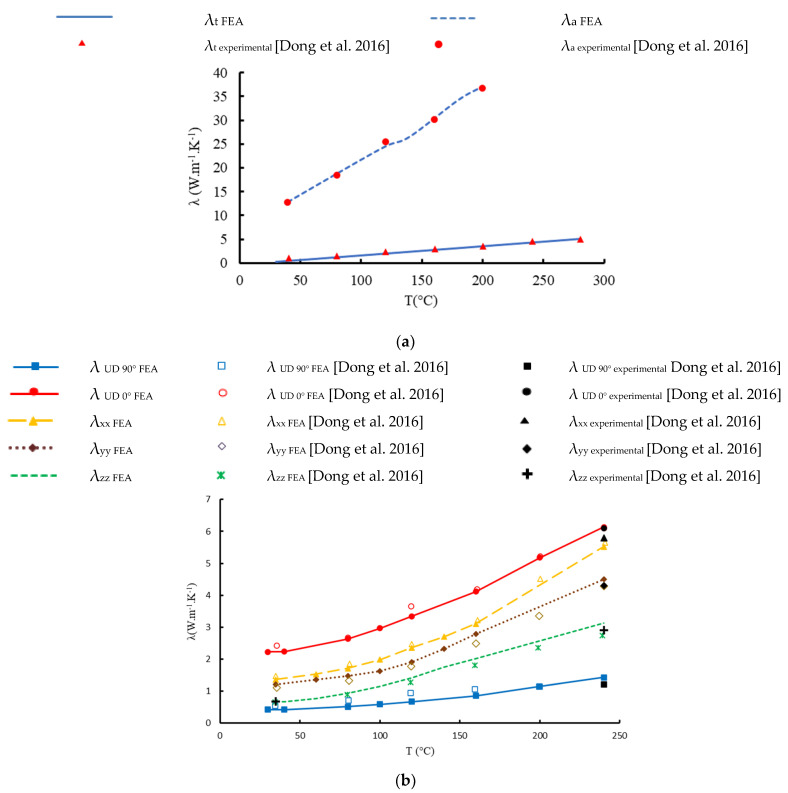
Thermal conductivity versus the temperature in the local directions [23]: (**a**) micro-scale (carbon fiber tow); (**b**) macro-scale: comparison of the UD lamina with the plain woven composite.

**Figure 7:**
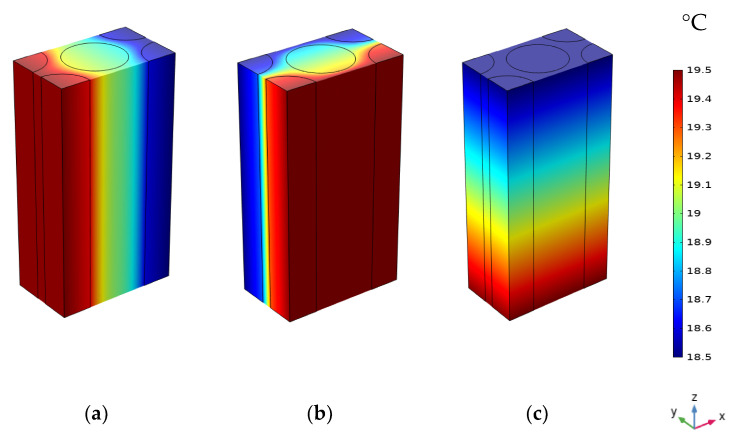
Temperature distributions of the yarn in the three local coordinate directions: (**a**) T_x_; (**b**) T_y_; (**c**) T_z_.

The expressions for the yarn’s axial and transverse thermal conductivities of the yarn for the Kulkarni and Brady model [27] are defined as follows:

(8) $$\lambda_{ya}=\lambda_{f∥}\;V_{fy}+\lambda_{m}\;(1-V_{fy})$$

(9) $$\lambda_{yt}=\lambda_{m}\;\left[\frac{\lambda_{f⊥}\;(1+V_{fy})+\lambda_{m}\;(1-V_{fy})}{\lambda_{f⊥}\;(1-V_{fy})+\lambda_{m}\;(1+V_{fy})}\right]$$

where λ_f║_, λ_f┴_, λ_m_ are respectively the TC along the axial direction of the fiber, the TC normal to the axial direction of the fiber, and the TC of the matrix. V_fy_ is the volume fraction of the fiber in the yarn. Table 4 shows the values of TCs computed with COMSOL Multiphysics compared to the values calculated using analytical models and referring to the experimental findings of Dong et al. [23] for a volume fraction in the yarns equal to 75.3%. A good agreement is observed with an error of less than 9%. The model of Kulkarni and Brady [27] is the closest to the actual experimental and numerical findings, with an error of less than 2.5%. As a result, this model can compute the yarns’ thermal conductivities made from any material at any temperature. Figure 6a shows a comparison of our findings with those of Dong et al. [23] for the axial and transverse thermal conductivities of yarns as a function of temperature for a fiber volume fraction of 75.3%. The TC is found to be a linear function of temperature, and the heat conductivity of the material is greater in the axial direction than in the radial direction. The distribution of the temperature within the micro-scale unit cell in three local directions is shown in Figure 7.

### 4.2. Full-Scale Validation

The thermal characteristics of the yarn obtained from unit cell 1 at different temperatures are included as input into unit cell 2. The FEA results are compared with the FEA values of Dong et al. [23] and with the experimental values at temperature T = 240 °C. The values are consistent with the FEA and experimental results of Dong et al. [23], as shown in Figure 6b. A comparison of the TC of woven composite and UD lamina can simply determine that the woven composite has better TC than 90°UD lamina and lower than 0°UD lamina when the heat path is known. Obviously, carbon fiber tows have superior TC compared to epoxy resin, especially through the axial direction. Therefore, heat will follow the path of the carbon fiber. The 0° and 90° lamina resemble a plain woven composite when they are combined. Thus, a simplification of plain-woven composites can be done by combining UD laminas. The decomposition method is further explained by Dong et al. [23].

## 5. Results and Discussion

### 5.1. Local Characteristics

**Figure 8:**
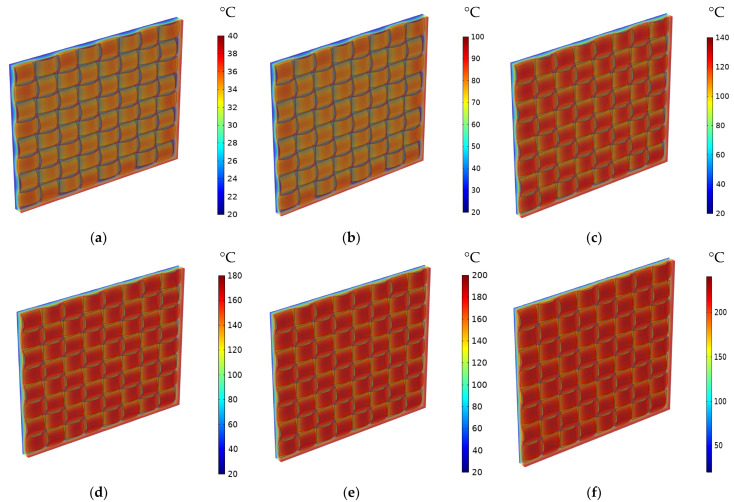
Distribution of the temperature for 1-layered composite for different set temperatures and at V_f_ = 32.56%: (**a**) T = 40 °C; (**b**) T = 100 °C; (**c**) T = 140 °C; (**d**) T = 180 °C; (**e**) T = 200 °C; (**f**) T = 240 °C.

**Figure 9:**
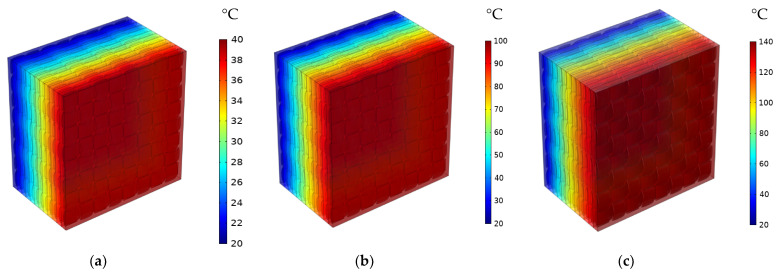

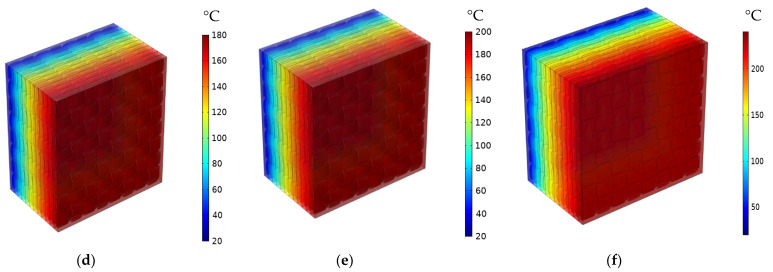
Distribution of the temperature for 12-layered composite for different set temperatures and at V_f_ = 32.56%: (**a**) T = 40 °C; (**b**) T = 100 °C; (**c**) T = 140 °C; (**d**) T = 180 °C; (**e**) T = 200 °C; (**f**) T = 240 °C.

In the macro-scale model, only heat transfer by conduction is considered. The latter depends basically on the TC of specific materials. Effective properties of the composite can be estimated based on the results concerning simulated temperature distribution or heat flux. The distribution of temperature for 1-layered and 12-layered composites for a different set temperatures is shown sequentially in Figure 8 and Figure 9.

**Figure 10:**
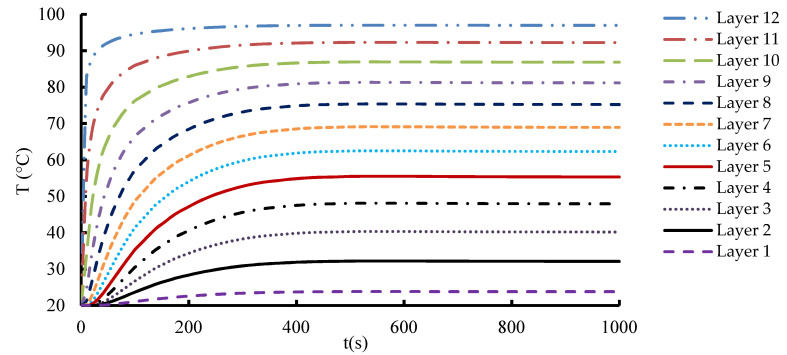
Temporal evolution of the temperature through all layers at T_h_ = 100 °C.

**Figure 11:**
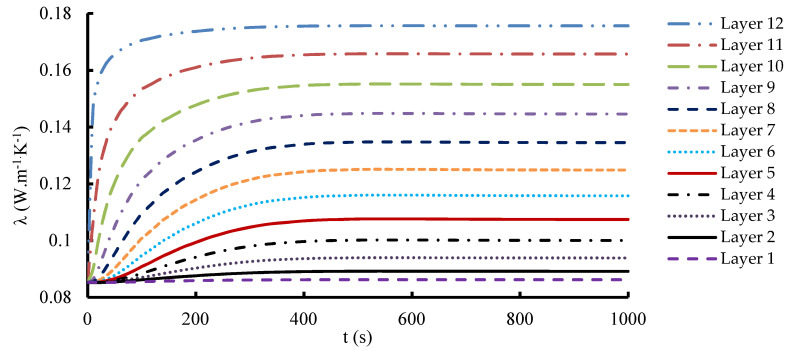
Temporal evolution of the thermal conductivity through all layers at T_h_ = 100 °C.

**Figure 12:**
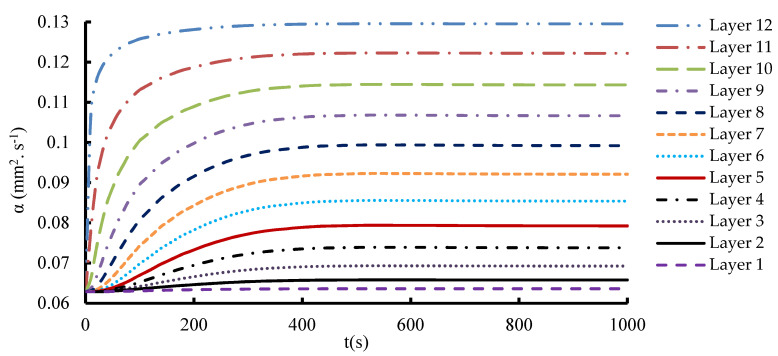
Temporal evolution of the thermal diffusivity through all layers at T_h_ = 100 °C.

In the 1-layered composite, the temperature distribution is typically more uniform across the entire composite and the temperature gradient within the composite is generally gradual. This is because the heat flows more easily in a single layer without significant interruptions or interfaces. In contrast, the 12-layered composite introduces additional interfaces between the layers. The presence of these interfaces can affect the temperature distribution within the composite. Depending on the thermal conductivity mismatch between the layers, there may be temperature variations at the interfaces due to thermal resistance. This can result in localized temperature gradients or hot spots near the interfaces. Figure 10, Figure 11 and Figure 12 serve to illustrate the temporal evolution profiles of temperature, TC, and thermal diffusivity across the entirety of the material’s layers, with a specific scenario in focus. This scenario entails a prescribed elevated temperature, denoted as T = 100 °C, and a fabric volume fraction of V_f_ = 32.56%.

Notably, layer 12, positioned as the exterior interface exposed to the elevated temperature, showcases distinct characteristics within this context. Anticipatedly, the curves for the specimens’ temperature progression reflect a swift initial ascent, followed by a gradual and stabilizing phase where the temperature attains a consistent value aligning with the volume-averaged representation of the applied elevated temperature. An observation of significance emerges as the temporal patterns of TC and thermal diffusivity mirror that of the temperature curve model. This concurrence underscores the intricate interplay between temperature, TC, and diffusivity. A discernible pattern surfaces in the transmission of heat from one layer to the next, leading to temperature differentials that oscillate and consequentially impact the TC within each layer. This dynamic interlayer interaction bestows unique thermal behaviors upon each layer. Consequently, the sequential layers each exhibit a distinctive TC value. Considering the composite’s homogeneity, wherein uniform layers comprise the composition, an inherent predictability arises regarding the effective thermal properties. In this context, the directional orientation of heat flux ceases to exert any influence on these properties. The inherent similarity in the composition and arrangement of layers thus bolsters the foreseeability of the composite’s thermal characteristics, even in the presence of varying heat flux directions.

The rate of change and the ultimate equilibrium values of TC can provide valuable information about the material’s ability to conduct and distribute heat over time. This information can be used to assess the composite’s transient thermal behavior, heat dissipation characteristics, and suitability for applications with varying temperature profiles. As evident from the graph, the TC exhibits a distinctive trend along the thickness of the composite. Starting from the outermost layer, which is typically dominated by the polymer matrix, we observe a gradual decline in TC. This reduction can be associated with the lower TC of the matrix compared to the carbon fibers. As we move deeper into the composite, closer to the central layers, where the carbon fibers are densely packed, a significant increase in TC is observed. This sudden change suggests that carbon fibers significantly contribute to improving heat conduction.

Thermal diffusivity is a parameter that reflects how quickly heat is conducted and diffused within a material, and in the context of CFRP composites, it plays a significant role in understanding heat transfer characteristics. Obviously, the thermal diffusivity increases as the temperature increases. The mean effective thermal diffusivity represents the composite’s ability to transport heat and is typically an average value. Variations in thermal diffusivity at different temperatures can reveal how the material’s heat conduction properties change with thermal stress. Understanding this distribution is essential for predicting how the composite will respond to temperature variations in real applications. Examining this distribution can provide insights into how heat is conducted through different layers of the composite at different temperatures. It illustrates the anisotropic characteristics of the material.

### 5.2. Effect of the Fiber Volume Fraction

**Figure 13:**
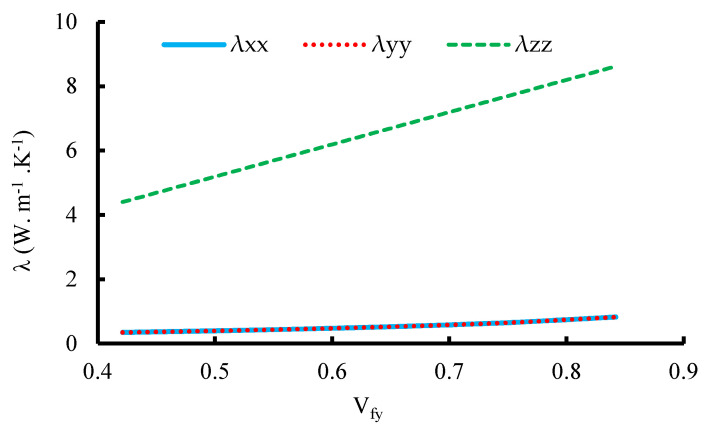
Thermal conductivity as a function of the volume fraction of fibers inside the yarn for unit cell 1.

The micro-scale RVE based primarily on the fiber content in the yarn V_fy_, which in this study is 75.3%, can be regarded as UD-fiber-reinforced composites. This scaled model’s thermal conductive behavior is investigated as a function of the thermal conductive parameters of the fibers and the resin. Figure 13 shows the relationship between the TC and the volume fraction of fibers at room temperature equal to T = 25 °C for the micro-scale model. Based on these findings, it is clear that the TC rises with the increasing volume fraction of the fibers inside the yarn. This can be explained by the increase in the fiber content and decrease in the resin content resulting in the increase in the TC of the fabric.

**Figure 14:**
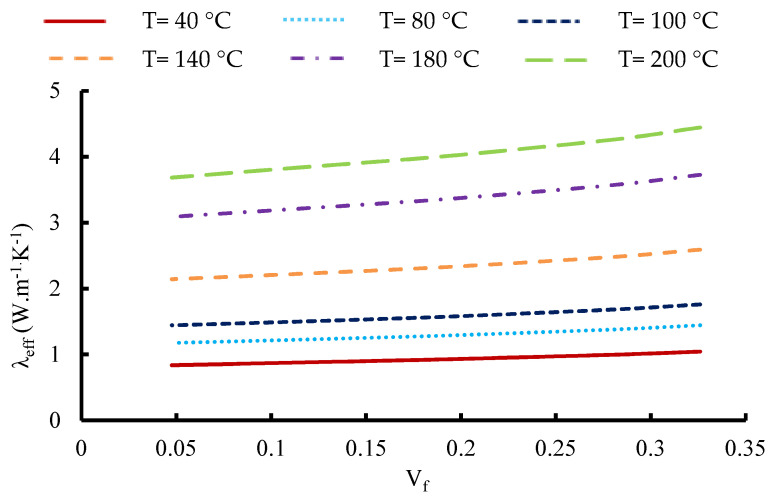
Variation in the effective thermal conductivity of the 12-layered composite versus fabric volume fraction for different set temperatures.

In Figure 14, a visual representation is provided, elucidating the variability in the ETC exhibited by the 12-layered composite. This variation is assessed with respect to alterations in the fabric volume fraction while accounting for different prescribed setting temperatures. Notably, a consistent trend becomes evident across the depicted scenarios. As foreseen, the observed TC exhibits a clear dependency on the fabric volume fraction, aligning with expectations surrounding this relationship. At lower temperatures (40 °C and 80 °C), the curves demonstrate a gradual rise in ETC as the proportion of fabric layers increases. This suggests an enhanced heat conduction through the fabric layers under these conditions. During intermediate temperatures (100 °C and 140 °C), the curves exhibit a steeper incline, indicating a more pronounced enhancement in ETC as the fabric volume fraction grows. This implies that fabric layers likely play a heightened role in facilitating heat conduction at these intermediary temperatures. Conversely, at higher temperatures (180 °C and 200 °C), the curves begin to level off, suggesting that the influence of fabric volume fraction on ETC diminishes as temperatures rise. This reduction in influence could be attributed to the increasing dominance of alternative heat transfer mechanisms at these elevated temperatures.

Some correlations of the TC versus the fabric volume fraction can be written as a linear function with a correlation coefficient equal to R^2^ = 0.98.

For T = 40 °C, it is possible to obtain the following expression:

(10) $$\lambda_{eff}=0.7437\;V_{f}+0.7921$$

while for T = 80 °C, it is:

(11) $$\lambda_{eff}=0.9703\;V_{f}+1.1136$$

for T = 100 °C:

(12) $$\lambda_{eff}=1.1481\;V_{f}+1.3688$$

for T = 140 °C:

(13) $$\lambda_{eff}=1.6226\;V_{f}+2.0383$$

for T = 180 °C:

(14) $$\lambda_{eff}=2.2929\;V_{f}+2.9476$$

and for T = 200 °C:

(15) $$\lambda_{eff}=2.724\;V_{f}+3.5197$$

### 5.3. Effect of Temperature

**Figure 15:**
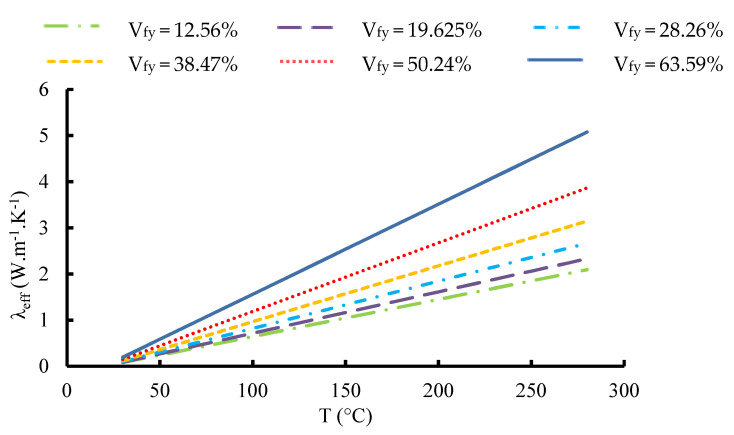
Variation in the effective thermal conductivity inside carbon tow as a function of temperature for different fiber volume fractions.

The temperature dependency of the TCs of the yarns has been empirically proven. Figure 15 depicts the effective thermal conductivities of a yarn estimated using a micro-scale unit cell. At low fiber volume fraction, the ETC demonstrates a relatively modest trend across temperatures, suggesting that the material’s heat conduction efficiency is hindered due to the scarcity of thermally conductive fibers. As the fiber content rises within the range of 19.625% to 50.24%, there is a consistent pattern of the ETC increasing with rising temperatures, indicating that a higher proportion of carbon fibers contributes to improved heat conduction capabilities. At the highest fiber volume fraction of 63.59%, the ETC reaches its peak value and maintains a steady trend across the temperature range, possibly indicating that the material’s heat conduction capacity has saturated due to the high concentration of thermally conductive fibers. The equation of the ETC as a function of the temperature and the volume fraction for carbon fiber may be written for the micro-scale model as well as the UD carbon fiber reinforced composite as follows:

(16) $$\lambda_{eff}=0.0195\;T+0.216\;V_{fy}-0.53$$

where T is the temperature and V_fy_ is the volume fraction of fibers inside the yarn. This equation can be used as an input for the macroscale model. It will facilitate the computation for this kind of material. A parametric sweep combining the two variables could be used directly by choosing for each variable an interval of values, which would give much more reasonable and effective results. It also helps to do optimization at the level of meso-microscopic scale, knowing that failure may develop gradually due to thermal loads without interference from mechanical stresses. In fact, thermal stress generated by temperature change can affect the stiffness and the strength of the material which can lead to material failure.

**Figure 16:**
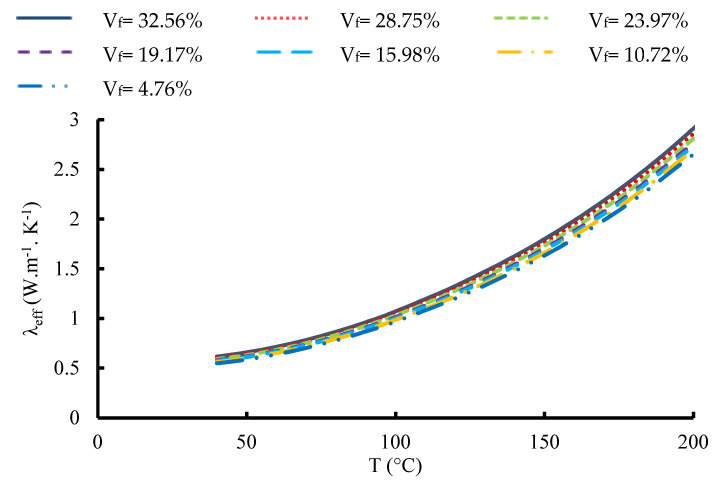
Variation in the effective thermal conductivity of 1-layered composite versus temperature for different fabric volume fractions.

**Figure 17:**
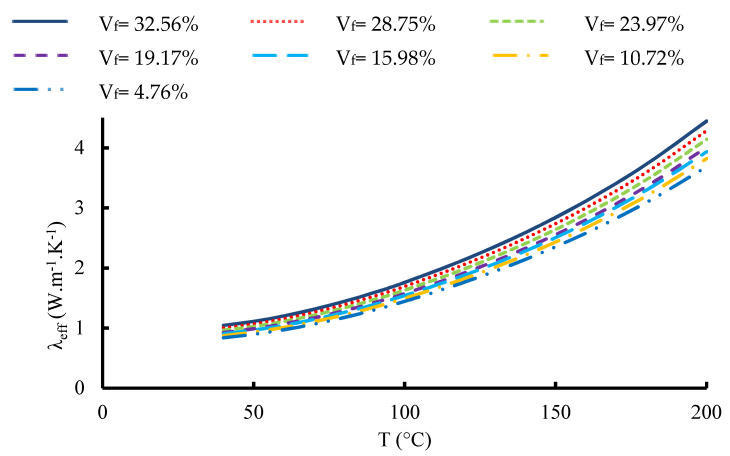
Variation in the effective thermal conductivity of 12 layered composite versus temperature for different fabric volume fractions.

Figure 16 and Figure 17 show the variation in the ETC of the 1-layered composite and the 12-layered composite, respectively, versus temperature at different volume fractions of fabric. For a volume fraction corresponding to 32.56% and for the 1-layered composite, the ETC increases from 0.6176 W.m^−1^.K^−1^ at 40 °C to 2.912 W.m^−1^.K^−1^ at 200 °C. For the 12-layered composite, it ranges from 1.0427 W.m^−1^.K^−1^ at 40 °C to 4.445 W.m^−1^.K^−1^ at 200 °C. For the single-layered carbon/epoxy composite, the correlation between the ETC and temperature at V_f_ = 32.56% can be written as:

(17) $$\lambda_{eff}=7\;10^{-5}\;T^{2}-0.002\;T+0.61$$

and for the 12-layered carbon/epoxy composite, it can be written:

(18) $$\lambda_{eff}=9\;10^{-5}\;T^{2}-0.001\;T+0.95$$

Those two correlations are second-degree polynomials, and they give an improved TC with a correlation coefficient equal to R^2^ = 0.999.

**Figure 18:**
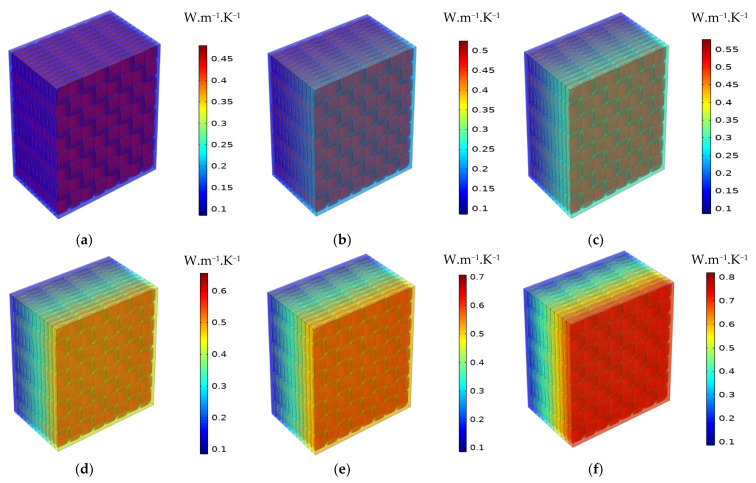
Distribution of the thermal conductivity through thickness for different temperatures V_f_ = 32.56%: (**a**) T = 40 °C; (**b**) T = 100 °C; (**c**) T = 140 °C; (**d**) T = 180 °C; (**e**) T = 200 °C; (**f**) T = 240 °C.

**Figure 19:**
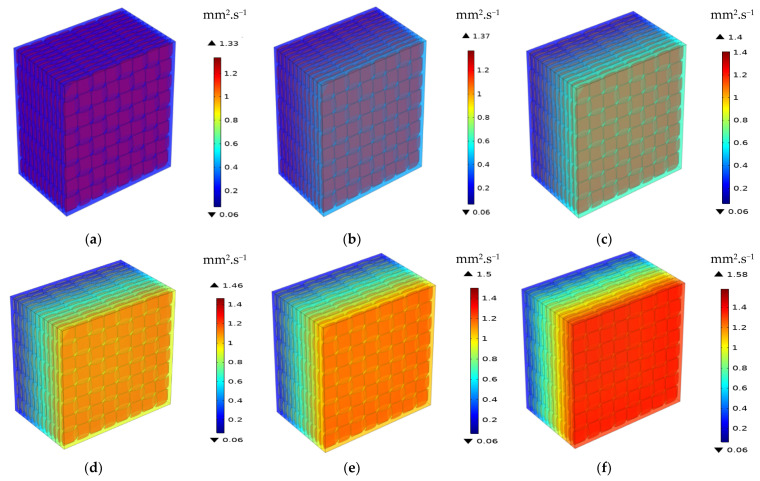
Distribution of the thermal diffusivity through thickness for different temperatures V_f_ = 32.56%: (**a**) T = 40 °C; (**b**) T = 100 °C; (**c**) T = 140 °C; (**d**) T = 180 °C; (**e**) T = 200 °C; (**f**) T = 240 °C.

As expected, TC increases as the volume fraction of the fibers rises. Also, it has been observed that it rises as the temperature increases. As a result, the TC varies proportionally to the temperature and fiber volume fraction. The same pattern can be noticed for thermal diffusivity and heat flux. Figure 18 and Figure 19 depict the distribution of the TC and the thermal diffusivity over thickness for various temperatures and V_f_ = 32.56%. The red zones represent the highest values, and the blue ones represent the lowest. The degradation between them shows that the TC and thermal diffusivity are dependent on temperature. Indeed, it propagates progressively as the temperature increases.

### 5.4. Effect of the Porosity of Matrix

The presence of voids inside the epoxy matrix is one of the internal flaws that might harm the CFRP composite. In fact, gradual failure occurs when voids are introduced into the microstructure due to a variety of reasons, including the curing process. In this part, the voids are supposed to be filled with air that has a TC equal to 0.026 W.m^−1^.K^−1^. To facilitate the simulation, homogenization theory is employed to estimate the TC on the micro- and macro-scale levels. The porosity ranges from 0 to 15%. Table 5 shows the variation in the TC of yarn for different porosities and fiber volume fractions. It can be observed that the axial and transversal thermal conductivities decrease as the porosity increases. The transverse TC decreases for low porosity values, with an estimated rate of 11.36%, 10.7%, and 8% for fiber volume fractions of 28.26%, 38.47%, and 63.59%, respectively. The proportion of reduction in TC drops as the fiber volume fraction increases. However, the porosity slightly affects the axial TC. In fact, it decreases by 0.55%, 0.35%, and 0.12% for fiber contents of 28.26%, 38.47%, and 63.59% respectively. Transverse TC is believed to be more vulnerable to the effect of porosity than axial TC. This may be explained by the fact that as the fiber content rises, carbon fiber becomes increasingly dominant in terms of heat conductivity. The axial TC of carbon fiber is higher and has a stronger impact on the ETC than the porous epoxy.

On the other hand, the impact of porosity on the ETC of woven composite and UD laminas as a function of temperature is reported in Table 6. Obviously, the TC decreases as the porosity increases. Also, it can be noted that the porosity has a growing impact on TC as the temperature rises. For instance, the rate of decrease accelerates from 6 to 9% for temperature rising from 30 to 240 °C for a porosity range of 0.05 to 0.15 for woven composite. Clearly, even with low porosity values, the TC decreases especially in the transversal direction, which could cause internal damage to the CFRP composite.

### 5.5. Effect of the Interphase Thickness

A crucial component of composites is the fiber-matrix interfacial region since it is where loads are transferred from the matrix to the fiber simultaneously. In composites, the fiber-matrix interfaces are made up of a coating layer made of one or more materials that are coated on the fiber, called interphase. Here, the impact of interphase thickness will only be applied to the microscale model. For the interphase, two materials were used: pyrolytic carbon and polypropylene. The thermal conductivities and densities of pyrolytic carbon are 3.5 W.m^−1^.K^−1^ and 1.4 g.cm^−3^, respectively, and 0.13 W.m^−1^.K^−1^ and 0.9 g.cm^−3^ for polypropylene at T = 25 °C. Several interphase thicknesses are selected, ranging from 0.1 to 0.4 µm. The influence of the interphase thickness on the TC of yarn for different volume fractions in the three local directions is presented in Table 7. It can be noted that the in-plane and out-of-plane thermal conductivities decrease with increasing interphase thickness. Taking, for example, fiber volume fraction equal to 63.59%, it can be seen that the axial TC slightly decreases as the interphase thickness increases with 1.15% for pyrolytic carbon and 0.05% for polypropylene when the thickness is equal to 0.1 µm. However, the transverse TC registered a massive drop with 65.48% for pyrolytic carbon and 56.81% for polypropylene. For ti = 0.1 µm, the transverse TC decreases rapidly, and the rate of decrease rises as the fiber volume fraction increases. For pyrolytic carbon, it decreases by 34.95% for V_fy_ = 28.26%, 54.98% for V_fy_ = 50.24%, and 65.48% for V_fy_ = 63.59%. For the polypropylene, the transverse TC registers a drop of 32.68% for V_fy_ = 28.26%, 49.55% for V_fy_ = 50.24%, and 56.81% for V_fy_ = 63.59%. On the other hand, the axial TC declines gradually and slowly, at a rate of around 0.05% to 0.08% for every 0.05 µm for polypropylene and less than 2% for pyrolytic carbon. It is possible to deduce that the interphase thickness affects the composite’s axial TC less than its transverse TC.

Similar to previous patterns, there is a clear anisotropic behavior, with different TCs along different axes. This indicates that heat conducts differently in various directions within the yarn material. An interesting observation is that the ratio of TCs (λ_zz_/λ_xx_ or λ_zz_/λ_yy_) tends to be significantly higher for pyrolytic carbon compared to polypropylene. This implies that pyrolytic carbon exhibits a stronger anisotropic behavior, with a more pronounced difference between TCs along different axes. These findings are valuable for material designers and engineers. They can guide decisions on selecting appropriate materials, fiber volume fractions, and interphase thicknesses to achieve desired TC profiles for specific applications. For instance, pyrolytic carbon might be preferred in applications where anisotropic thermal behavior is advantageous.

### 5.6. Effect Mechanism of Thermal Performance

In this section, the effect mechanism of thermal performance in the plain-woven CFRP composites based on the simulation results is explored. In fact, the simulation results provide valuable insights into the thermal performance of the composite. The findings reveal that the temperature and the fiber volume fraction exhibit the highest impact on thermal conductivity. Those two factors have important roles in enhancing the heat transmission, which can lead to reduced compression work during the filling process, resulting in substantial energy saving. Furthermore, the interphase thickness plays a crucial role in minimizing energy loss and improving heat insulation. In fact, the reduction in thermal conductivity leads to a reduction in heat loss, which contributes to improved energy efficiency during storage and transportation of hydrogen. It is worth noting that the porosity or the voids inside the matrix, while having the least impact, should still be considered in optimizing the thermal performance of the composite. Indeed, reducing the porosity level to below a certain threshold can help minimize heat loss caused by air gaps or voids.

The principles of optimization theory can be applied to further enhance heat transmission and insulation. Techniques such as Design of Experiments and response surface methodology can be utilized to identify the optimal combination of temperature, fiber volume fraction, interphase thickness, and porosity. These optimization approaches can help determine the specific parameter values that maximize heat transmission efficiency and minimize energy loss within the hydrogen storage tank. By implementing these optimization strategies and achieving the targeted improvements, significant energy savings and enhanced thermal performance in hydrogen storage systems can potentially be achieved. It is important to note that the actual values for heat transfer efficiency and heat loss can vary significantly depending on the system’s characteristics and the specific optimization strategies employed. Therefore, conducting detailed simulations or experiments tailored to the specific system is crucial for obtaining accurate and reliable results.

## 6. Conclusions

A multiscale approach for evaluating the effective thermal properties of plain weave CFRP composites is described in this study, emphasizing the interplay of key factors. The results demonstrate that both fiber volume fraction and temperature exert significant influence on the TC of the composite. Notably, the interphase thickness emerges as a critical factor, closely following the impact of fiber volume fraction and temperature. Specifically, for a fiber volume fraction in the yarn of 63.59%, a significant reduction in transverse thermal conductivity for pyrolytic carbon (65.7%) and polypropylene (64%) when the interphase thickness increased to 0.4 µm is observed. Nevertheless, a slight reduction in the axial direction occurs, with less than 3% for pyrolytic carbon and 0.05% for polypropylene. These numerical findings highlight the pronounced influence of interphase thickness on the composite’s thermal properties. Moreover, our investigation quantifies the impact of matrix porosity on TC, revealing a relatively slighter effect, particularly within the porosity range of 5 to 15%. This quantitative insight provides a nuanced understanding of how matrix porosity decreases TC. Knowing these points highlights its importance in preventing or delaying micro-cracking in the matrix, which can affect material failure and fatigue performance under thermal loads. In essence, this study offers comprehensive insights into the factors influencing thermal properties, providing valuable considerations for optimizing thermal performance in industrial applications.

## Figures and Tables

**Table 1:** Thermal properties of materials at room temperature (T = 25 °C) [23].

Materials	Density (g.cm^−3^)	Thermal Conductivity (W.m^−1^.K^−1^)		Specific Heat Capacity (J.kg^−1^.K^−1^)
		$\lambda_{∥}$	$\lambda_{⊥}$	
Carbon fiber (T700S-12K)	1.8	10.2	1.256	750
Epoxy resin (JC-02A)	1.13	0.18	0.18	1200

**Table 2:** Parameters of the meso-scale unit cell model [23].

RVE (mm)			Yarn (mm)				
d	h	V_fy_	a	b	e	V_f_	V_y_
8	1	75.3%	1.8	0.2	0.4	32.56%	43.25%

**Table 3:** Meshing.

Mesh Type	Extra Coarse	Normal	Finer	Extra Fine	Extremely Fine
Number of Elements	963	4903	15,492	47,473	259,950
λ_xx_	0.513	0.506	0.505	0.505	0.505
λ_yy_	0.503	0.505	0.504	0.504	0.504
λ_zz_	6.439	6.507	6.508	6.508	6.508
Time of computation	1 min 15 s	1 min 22 s	3 min 30 s	11 min 34 s	14 min 45 s

**Table 4:** Thermal conductivities of the yarns at room temperature (T = 25 °C) at V_fy_ = 75.3% compared to the analytical models and experimental results.

		Models			Experimental [23]	This Study
		Parallel	Pilling	Kulkarni and Brady		
λ_ya_		7.725	-	7.725	-	7.715
λ_yt_		-	0.723	0.646	0.664	0.662
Error (%)	λ_ya_	0.129	-	0.129	-	
	λ_yt_	-	8.43	2.416	0.346	

**Table 5:** Thermal conductivity of yarn for different fiber volume fractions and porosities.

V_fy_	28.26%			38.47%			63.59%		
φ	λ_xx_	λ_yy_	λ_zz_	λ_xx_	λ_yy_	λ_zz_	λ_xx_	λ_yy_	λ_zz_
0	0.2772	0.2772	3.0126	0.3272	0.3272	4.0355	0.5273	0.5273	6.5543
0.01	0.2752	0.2752	3.0115	0.3250	0.3249	4.0345	0.5246	0.5246	6.5538
0.015	0.2741	0.2741	3.0109	0.3238	0.3238	4.0340	0.5232	0.5232	6.5535
0.03	0.2710	0.2710	3.0093	0.3204	0.3203	4.0326	0.5191	0.5191	6.5526
0.05	0.2668	0.2668	3.0070	0.3157	0.3157	4.0307	0.5136	0.5136	6.5515
0.1	0.2563	0.2563	3.0015	0.3041	0.3041	4.0260	0.4994	0.4994	6.5487
0.15	0.2457	0.2457	2.9960	0.2922	0.2922	4.0212	0.4847	0.4847	6.5459

**Table 6:** Thermal conductivity of UD lamina and plain woven composite for different porosities and temperatures at V_f_ = 32.56%.

φ	0.05			0.1			0.15		
T_h_ (°C)	UD 0°	UD 90°	λ_eff_	UD 0°	UD 90°	λ_eff_	UD 0°	UD 90°	λ_eff_
30	2.1831	0.3726	0.5856	2.1831	0.3726	0.5662	2.1831	0.3726	0.5467
40	2.1834	0.3729	0.6143	2.1834	0.3729	0.5936	2.1834	0.3729	0.5729
80	2.5671	0.4496	0.8715	2.5664	0.4494	0.8386	2.5657	0.4492	0.8057
100	2.8883	0.5138	1.0856	2.8875	0.5137	1.0425	2.8865	0.5136	0.9992
120	3.2549	0.5826	1.3539	3.2539	0.5825	1.2977	3.2527	0.5823	1.2413
160	3.9856	0.7193	2.0536	3.9855	0.7194	1.9634	3.9853	0.7194	1.8728
240	5.7295	1.0202	3.0371	5.7287	1.0199	2.8988	5.7277	1.0195	2.7601

**Table 7:** Yarn thermal conductivity for various fiber volume fractions and interphase thicknesses.

		Pyrolytic Carbon			Polypropylene		
t_i_ (µm)	V_fy_	28.26%	50.24%	63.59%	28.26%	50.24%	63.59%
0	λ_xx_	0.2772	0.4018	0.5273	0.2772	0.4018	0.5273
	λ_yy_	0.2772	0.4018	0.5273	0.2772	0.4018	0.5273
	λ_zz_	3.0126	5.2162	6.5543	3.0126	5.2162	6.5543
0.1	λ_xx_	0.1803	0.1809	0.1820	0.1866	0.2027	0.2277
	λ_yy_	0.1803	0.1809	0.1820	0.1866	0.2027	0.2277
	λ_zz_	2.9591	5.1243	6.4786	3.0101	5.2119	6.5508
0.15	λ_xx_	0.1802	0.1806	0.1813	0.1845	0.1956	0.2133
	λ_yy_	0.1802	0.1806	0.1813	0.1845	0.1956	0.2133
	λ_zz_	2.9401	5.0911	6.4511	3.0089	5.2098	6.5491
0.2	λ_xx_	0.1801	0.1804	0.1810	0.1834	0.1919	0.2056
	λ_yy_	0.1801	0.1804	0.1810	0.1834	0.1919	0.2056
	λ_zz_	2.9244	5.0640	6.4281	3.0076	5.2077	6.5474
0.25	λ_xx_	0.1801	0.1803	0.1808	0.1828	0.1896	0.2007
	λ_yy_	0.1801	0.1803	0.1808	0.1828	0.1896	0.2007
	λ_zz_	2.9112	5.0411	6.4084	3.0064	5.2056	6.5457
0.3	λ_xx_	0.1801	0.1803	0.1807	0.1823	0.1881	0.1975
	λ_yy_	0.1801	0.1803	0.1807	0.1823	0.1881	0.1975
	λ_zz_	2.9000	5.0215	6.3915	3.0053	5.2036	6.5441
0.35	λ_xx_	0.1801	0.1802	0.1806	0.1820	0.1870	0.1951
	λ_yy_	0.1801	0.1802	0.1806	0.1820	0.1870	0.1951
	λ_zz_	2.8904	5.0046	6.3768	3.0041	5.2016	6.5424
0.4	λ_xx_	0.1801	0.1802	0.1805	0.1817	0.1861	0.1932
	λ_yy_	0.1801	0.1802	0.1805	0.1818	0.1861	0.1932
	λ_zz_	2.8820	4.9900	6.3640	3.0029	5.1996	6.5408

## Data Availability

The authors declare that the data supporting the findings of this study are available within the paper.

## Nomenclature

T	Temperature, °C
T_c_	Cold temperature, °C
T_h_	Hot temperature, °C
a	Long axis of the yarn, m
b	Short axis of the yarn, m
d	Width and length of the meso-scale unit cell model, m
e	Yarn spacing, m
h	Height of the meso-scale unit cell model, m
l	Length of the yarn, m
q	Density of heat flow, W.m^−2^
V	Volume fraction
t	Time, s
t_i_	Interphase thickness, m
**Symbols**	
α	Thermal diffusivity, m^2^.s^−1^
λ	Thermal conductivity, W.m^−1^.K^−1^
φ	Porosity
ΔT	Temperature difference, K
∇ T	Temperature gradient, K.mm^−1^
ρ	Density, kg m^−3^
C_p_	Specific heat capacity, J.kg^−1^.K^−1^
**Subscripts**	
f	Carbon fibers in plain woven composite
m	Epoxy resin matrix
eff	Effective
y	Yarn
fy	Fiber in yarn
xx	Across the heat flow path
yy	Normal to the heat flow path
zz	Out-of- plane direction
	Parallel to the axial direction of the fiber
┴	Normal to the axial direction of the fiber
ya	Axial direction of the yarn
yt	transverse direction of the yarn
in	In-plane direction
out	out-of-plane direction (thickness)
**Abbreviation**	
CFRP	Carbon-fiber-reinforced polymer
ETC	Effective thermal conductivity
FEA	Finite Element Analyses
RVE	Representative Volume Element
TC	Thermal conductivity
UD	Unidirectional lamina
